# Innovations in public health surveillance: updates from a forum convened by the WHO Hub for Pandemic and Epidemic Intelligence, 2 and 3 February 2022

**DOI:** 10.2807/1560-7917.ES.2022.27.15.2200302

**Published:** 2022-04-14

**Authors:** Technical contributors to the Pandemic and Epidemic Intelligence Innovation Forum February meeting and report, Technical contributors to the Pandemic and Epidemic Intelligence Innovation Forum February meeting and report, Oliver Morgan, Isabel Redies, Zoila Beatriz Leiva Rioja, John Brownstein, Dylan George, Josie Golding, Johanna Hanefeld, Peter Horby, Christopher Lee, Danil Mikhailov, Wolfgang Philip, Samuel Scarpino, Sofonias Kifle Tessema, Chikwe Ihekweazu

**Affiliations:** 1 The members of this group are listed under Collaborators.

**Keywords:** infectious diseases, surveillance, pandemic preparedness, technology, innovation, collaboration, capacities

In the 2 years since the emergence of severe acute respiratory syndrome coronavirus 2 (SARS-CoV-2) there has been an unprecedented collective effort from the academic, public, and private sectors to advance surveillance for pandemic preparedness and response. The coronavirus disease (COVID-19) pandemic has created momentum that will define the future of public health intelligence. On 2 and 3 February 2022, the World Health Organization (WHO) Hub for Pandemic and Epidemic Intelligence convened a meeting of a small group of surveillance innovators to share insights and approaches about their initiatives and future directions. The meeting served as an opportunity for participants to share updates about their work, to explore potential for collaboration, exchange ideas, cross-fertilise our work and discuss challenges in the field of surveillance. Although the group of attendees was not geographically representative of the global surveillance community, the meeting was the first in a planned series of exchanges convened by the WHO Pandemic Hub that will generate dialogue among global thought leaders and new voices in the surveillance community. In this first convening we discussed several themes, including what is meaningful collaboration for success; how to bring the public back into public health; what are individual-centred approaches; how new kinds of data have new privacy concerns; how government structures affect the functioning of surveillance systems; how to inform the decisionmaking process; cross-scaling and down-scaling tools and technologies; investing in human talent and future practitioners; and achieving sustainability into surveillance. In this meeting report, we summarise the discussions on innovations in public health surveillance and provide a list with references and links to the organisations and initiatives represented at the meeting.

## Meaningful collaboration for success

Research before and during the COVID-19 pandemic has suggested that trust (interpersonal and between organisations) is strongly associated with implementation of public health guidance, which can lead to better containment of outbreaks [1-5]. Therefore, there is an urgent need to build a trust architecture [6] between individuals, governments, the private sector, academia and non-governmental organisations (NGOs) that allows for meaningful collaboration. This trust architecture can, in turn, enhance the transparent exchange of information. However, the latter will be achieved only if information exchange results in resourceful collective action and global solidarity instead of restrictive measures and penalties. Further, surveillance efforts ought to be seen as a matter that goes beyond the health sector and one that requires close collaboration with other sectors. Having in place truly multidisciplinary teams with social and data scientists, communicators, user experience and user interface experts, technologists and more established public health experts is critical to ensure timely data availability.

The Robert Koch Institute (RKI) and the Health Emergency Preparedness and Response Authority (HERA) of the European Commission both presented their approaches to foster trust and collaboration. Throughout 2022, the RKI will undertake a landscaping exercise to gain a better understanding of the forecasting and data value chain, as well as a clear sense of which areas can benefit from more support in terms of functionality and capacity. This consultation will be carried out at global, regional and national levels, with a strong focus on the latter, aiming at identifying gaps and better practices that will serve as inputs for the development of a strategy. Representatives of HERA pointed out that a key aspect of their work around pandemic preparedness and response will be to engage and cooperate closely with partners from the European Union and beyond. Through this cooperation, HERA aims to ensure availability and access of critical medical countermeasures, address global supply chain bottlenecks and facilitate information, knowledge, and data sharing.

## Bringing the public back into public health

The containment of infectious disease outbreaks depends significantly on individuals’ health-seeking behaviours, knowledge, and attitudes towards public health measures. Insights into behavioural data are, therefore, a crucial aspect of pandemic preparedness and need to be effectively communicated to decisionmakers. However, the ability to quickly and routinely assess the population’s attitudes related to information about health emergencies and public health measures remains a challenge for the global pandemic surveillance community. Participatory approaches, like crowdsourcing, can be an effective way to engage citizens in the generation of such insights and “to put the public back into public health”. Outbreaks Near Me is an online platform where over six million people are actively engaged in providing information about COVID-19 symptoms, healthcare utilisation and vaccination uptake. The platform also collects data on rapid tests and feeds them back into local and national public health institutions. It therefore complements existing surveillance systems by capturing data on symptomology and vaccine effectiveness that does not require interaction with the healthcare system. Additionally, partnering with Momentive.ai, the platform hosts large-scale online surveys that generate real-time data on health-seeking behaviours and acceptance of public health measures, such as mask-wearing and regular testing. Their surveys on vaccine uptake, vaccine acceptance and differences in demand for vaccines from different manufacturers have directly informed decisionmaking processes around immunisation practices at the United States Centers for Disease Control and Prevention (US CDC). The representatives of participating initiatives agreed that there is a great need for the development of further tools that provide such actionable insights.

## Individual-centred approach

There was a common acknowledgement among representatives of participant organisations that individuals, whether they are public health officials, community leaders or community members, need to be at the centre of pandemic preparedness and response efforts. Organisations like the Pandemic Prevention Institute (PPI) at The Rockefeller Foundation are taking a user-centred design approach to build tools for public health decision support. By building profiles and conducting interviews with individuals they seek to empower, the PPI has gained insights into these individuals' behaviours, concerns, pain points and needs and has refined its tools with these insights. In addition, it is possible to learn more about what kinds of data and data sources (e.g. government, NGOs, multi-lateral, private sector, etc.) are most trusted. An example of the initiative’s work is the COVID-19 Risk Estimator, which estimates the probability of one COVID-19–positive person being at an event. Such tools can be used for all sorts of decisionmaking processes around mitigation measures at the community, hospital, household, and individual levels.

Further, information from patients is critical both to patient care and epidemic response. Patient information and samples enable the characterisation of pathogens and the determination of infectious tissues, potential transmission routes, risk groups and other key infectious disease parameters that inform public health measures. The International Severe Acute Respiratory and Emerging Infection Consortium (ISARIC) has played a key role in this topic through the generation and dissemination of patient-based research for emerging infectious diseases and epidemic diseases. The benefits of accessing clinical data have been recognised in various contexts. During the meeting, three country examples were brought forth: South Africa and the UK have provided the world with a large share of the information on variants during the COVID-19 pandemic, and in the United States the US CDC led the tracking of a cohort of individuals through time to understand the clinical outcomes of those infected with Delta vs Omicron, thus providing estimates of clinical severity to decisionmakers [7]. Despite the relevance of both behavioural and clinical data, access to this type of information remains a challenge for many countries.

## New kinds of data raise new privacy concerns

Ensuring privacy is a critical part of building trust. Currently, the landscape of laws, treaties and regulations for existing data and the evaluations of these by countries and international organisations are well known among the members of the surveillance community. Nevertheless, many privacy problems remain unsolved. Further, it is of the utmost importance to anticipate the challenges that will arise from new kinds of data and new ways to generate insights, such as new privacy risks, and risks for abuse and misuse of data. Legal issues around data sharing, that is, having clarity on how different datasets can be put together, how they can be combined into products and how they can be distributed, may turn out to be the biggest challenge of all. Hence, it is important to set clear legal standards. However, not all new types of new data for surveillance are associated with privacy concerns. Wastewater samples, for instance, can provide useful non-personally identifiable information for early warning systems to inform public health measures. Since individuals can shed SARS-CoV-2 in their faeces even if they are asymptomatic, an increase in viral levels in wastewater can predict where the number of cases will rise.

Exploring how technologies such as differential privacy, privacy-distributed machine learning and others can be of assistance in this matter is vital. Currently, many organisations are putting in place initiatives in this regard. As part of its activities for this year, data.org is running a call for a Challenge fund. This will be directed at innovations around privacy-preserving technologies for epidemiological modelling. The Computational Epidemiology Laboratory at Boston Children’s Hospital, for instance, launched a project together with Google that investigates the use of applications on Android phones that allow individuals to report on their behaviours and symptoms, while the data resides on their phones. With the use of federated analytics, aggregate data, and differential privacy assessments to modify the data, there is no possibility to use the data to identify individuals.

## Government structures affect the functionality of surveillance systems

Many representatives alluded to the fact that different government structures affect how surveillance systems can be developed and operated. Surveillance systems need to be tailored according to the political and structural realities of each environment. Colleagues from the Africa Centres for Disease Control and Prevention - Africa Pathogen Genomics Initiative (PGI), who seek to build a continent-wide Data Management and Exchange Platform, are faced with political and policy-related challenges. In many government systems, data governance, data ethics, data access, security, and sharing/access protocols are limited. Further, there are cumbersome government approval processes and a risk of sharing data without permission from relevant government offices.

Nationwide programs can also face policy-related challenges. For the work of the new US CDC Center for Epidemic Forecasting and Outbreak Analytics (CFA), for instance, the US-wide Data Modernization Initiative will be critical to their success. The Data Modernization Initiative seeks to make data flow more effectively within the US from different parts of the healthcare system to public health institutions at the speed of need. At the CFA, a specialised team of data analysts will focus on how this national technology architecture could be structured. This includes questions, for instance, about which kind of analytical capabilities should be pursued in a centralised or decentralised way. Throughout the meeting, it became clear that further exchanges and discussions are needed on the opportunities and challenges associated with different forms of government structures and their impact on surveillance systems.

## Informing the decisionmaking process

Providing actionable insights to policymakers to guide adequate policy response remains a huge challenge. The COVID-19 pandemic has laid bare many of the deficiencies in using data to inform public health policy decisions. At the same time, this pandemic represents a huge opportunity, since decisionmakers have become more familiar with some of these issues. Overall, it was agreed that there is a need for technology capability and simplification of processes that allow information to support timely and actionable risk communication and decision. Communication between relevant stakeholders should be carried out in a much more streamlined and effective way, allowing for a feedback loop between providers of data and decisionmakers that informs how data have been used and what decisions have been made. Although the relevance of a communication lens during interactions with decisionmakers is usually acknowledged, the incorporation of a contextualisation lens is also critical. The provision of data needs to be complemented by information on the intended and unintended consequences in the domains of behavioural science, economy, and security events, among others. In this way, data should inform the next policy steps while disease control efforts are balanced with the societal and economic costs of each context.

Systematic processes that contemplate the involvement of decisionmakers in daily surveillance activities ought to have predominance over passive models of just sending out information. Some examples and tools around this are the COVID-19 live dashboard implemented by ISARIC, which allows decisionmakers such as a Chief Medical Officer to see data updated by clinicians in real time and informs the government’s weekly scientific advisory group meetings; the scenario modelling and forecasts that will be a key focus area at the CFA; and the daily meetings of the Epidemic Intelligence Committee established by the Nigeria Centre for Disease Control (NCDC), as part of the implementation of the 7–1-7 approach proposed by Resolve to Save Lives [8]. The 7–1-7 framework is proving to be a strong metric that highlights the importance of measurement of the timeliness of decisionmaking for accountability and advocacy. It emphasises the need to go beyond just disease detection and to take prompt actions with clear goals for all emerging threats.

## Cross-scaling and down-scaling tools and technologies

The impact of epidemics and pandemics differs between communities. To apply effective COVID-19 containment measures, local decisionmakers need to be provided with a context-specific analysis and forecasts of pandemic and epidemic situations. One persistent challenge for the global surveillance community is the use and adaptation of technologies and tools across different geographies and scales. How can solutions that were built at a global or national level be applied to a local scale and vice versa? Meeting participants noted their organisation’s intention to transfer technologies and tools that have proven to be robust at one scale to another scale. Global.Health, for instance, created an open-source platform that is focused on the collection, secure data management and sharing of individual de-identified epidemiological case data. The platform aggregates the data with spatial and temporal detail and shows the rapidly evolving pandemic situation at a global level on their online dashboard. In cooperation with The Rockefeller Foundation, the initiative now works on creating a platform that has decentralised data source and storage to enable local decisionmakers to work with local data at a local scale.

The attendees discussed the transfer of analytical tools and technologies that effectively informed decisionmaking in one country or city to another local context. There was a shared intention to exchange tools across regions and to empower each other to adapt available technologies to context-specific needs. The Covid-19 Risk Estimator by the Pandemic Prevention Institute, for instance (mentioned above), was taken up by colleagues in Mexico who adapted the tool to their regional context. However, examples like these remain anecdotal. The use of instruments across different geographies still involves multiple challenges that call for further discussions within the surveillance community.

## Investing in human talent and future practitioners

The future of pandemic preparedness and response does not depend only on the creation of effective technologies for data exchange and analysis. It also depends on human talent and a concerted effort to train a “pandemic and epidemic intelligence workforce”. Particularly in lower- and middle-income countries, there is a need to build capacity to improve data systems and to analyse and translate data into actionable insights for public health decisionmaking. Many countries experience a scarcity of experienced technical experts who can be recruited by the public sector. There are also major gaps in subnational public health and clinical staff, who are critical for disease detection, response, and case management. It is therefore crucial to build the capacities of laboratory technologists and data scientists who can apply, maintain, and adapt technologies and tools to effectively inform their community’s decisionmakers. The Capacity Accelerator Network by data.org works with multiple partners and funders to set up programs to train data scientists with advanced technical skills and an interdisciplinary understanding of factors contributing to a health emergency. The initiative cooperates with local academic and public institutions and social impact organisations, as they are best equipped to tailor (and host) training programmes to educate the workforce they will likely employ. To ensure the sustainability of capacity-building efforts, teachers and trainers need to be trained to educate the new generation of data scientists for pandemic intelligence.

## Ensuring the sustainability of our work

The COVID-19 pandemic has created a window of opportunity for the global surveillance community as it has elevated the importance of pandemic preparedness and response in an unprecedented way. To keep the world safe and to avert and manage public health risks, the current public and political awareness of the devastating consequences of unabated health crises should not be lost. Many attendees made an analogy to the global weather forecasting system to express the attention/significance they would like pandemic prevention to assume in the long term. For the general public, funders and donors, and local, national, and global decisionmakers, the value of surveillance systems should be as clear as the value of a weather forecast. This understanding is a prerequisite for effective pandemic management with seamless cooperation between multiple surveillance systems, private sector companies, government agencies, and the participation of citizens.

In this context, one challenge for the public health intelligence space is to secure sustainable funding. Over the past 2 years, there has been a great stream of resources pouring into public health intelligence and information technology for surveillance and analytics. However, as the COVID-19 pandemic evolves, funding streams are already diminishing. Often, in the face of health emergencies, there is interest in funding the launch of innovative technological projects for immediate emergency relief. But it is often challenging to secure funds for the long-term support, maintenance and development of initiatives and newly created (data) infrastructures in this field. Some large tech companies have already disbanded specialised units that were set up to support public health surveillance and management of the pandemic. At the meeting, several approaches to securing funding were discussed. Multiple initiatives recommended a diversified funding strategy, where several funders from the public, philanthropic and private sector jointly contribute to the launch and long-term maintenance of projects. The EPIVERSE initiative by data.org which, for instance, constitutes a collaboration of funders including philanthropic organisations such as The Rockefeller Foundation, the Wellcome Trust, and tech companies that jointly fund initiatives to create and maintain a suite of open-source tools for epidemiological analytics and to build capacities around their application.

Attendees acknowledged that to sustain public, political and donor support, the surveillance community needs to consistently prove the robustness of their work. Similar to how weather forecasting and the ability to guide public response to weather events has improved since the 1960s, the robustness of epidemic and pandemic forecasting models and health risk communication needs to evolve significantly in the coming decades. To prove and ensure the robustness of surveillance technologies, there is a need for clear operational metrics that guide the development of analytical models and tools, and that monitor and evaluate their effectiveness, discarding them if they do not prove useful.

## Next steps

Based on the success of this meeting, the WHO Pandemic Hub established the Pandemic and Epidemic Intelligence Innovation Forum which will convene regular quarterly meetings with international domain experts and thought leaders. Throughout every meeting, we will discuss a specific topic of relevance to the future of pandemic and epidemic intelligence and explore collaboration opportunities. The next meeting will take place on 12 May 2022 and discuss ways of operationalizing data aggregation and linkage, the analytical value of data aggregation and the continued and open access to (non-)health data for pandemic and epidemic management. As the community of partners participating in the Forum continues to grow, we expect it to have greater engagement with a diverse global surveillance community (Box).

Box **Initiatives discussed at the forum, in order of appearance in the meeting report**

- Robert Koch Institute (RKI), landscaping exercise. https://www.rki.de/
- Outbreaks Near Me. https://outbreaksnearme.org/us/en-US
- Momentive, Agile Experience Management Solutions. https://www.momentive.ai/en/?ut_source2=en%2Fsolutions
- The Rockefeller Foundation, Pandemic Prevention Institute. https://www.rockefellerfoundation.org/pandemicpreventioninstitute/
- Kaitlyn Johnson, Holiday risk estimator: Likelihood that COVID-19 will be present at a holiday gathering. https://medium.com/@kjohnson_15125/holiday-risk-estimator-555e28b03afd
- International Severe Acute Respiratory and emerging Infections Consortium (ISARIC). https://isaric.org/
- Centers for Disease Control and Prevention (CDC), National Wastewater Surveillance System (NWSS). https://www.cdc.gov/healthywater/surveillance/wastewater-surveillance/wastewater-surveillance.html
- data.org, Inclusive Growth and Recovery Challenge. https://data.org/initiatives/challenge/
- Computational Epidemiology Laboratory. https://compepi.org/
- Africa Centers for Disease Prevention and Control (CDC), Institute of Pathogen Genomics (IPG). https://africacdc.org/institutes/ipg/
- CDC, New Disease Forecasting Center. https://www.cdc.gov/media/releases/2021/p0818-disease-forecasting-center.html
- ISARIC, COVID-19 Analysis Report. https://livedataoxford.shinyapps.io/CovidClinicalDataDashboard/
- Resolve to Save Lives. https://resolvetosavelives.org/
- Global.health, COVID-19 Map. https://map.covid-19.global.health/country
- data.org, Capacity Accelerator Network (CAN). https://data.org/initiatives/capacity/
- data.org, Epiverse: Distributed Pandemic Tools Programme. https://data.org/initiatives/epiverse/>
- The Rockefeller Foundation. https://www.rockefellerfoundation.org/
- Wellcome Trust. https://wellcome.org/
